# Biomarker Predictors of Clinical Efficacy of the Anti-IgE Biologic Omalizumab in Severe Asthma in Adults: Results of the SoMOSA Study

**DOI:** 10.1164/rccm.202310-1730OC

**Published:** 2024-04-18

**Authors:** Ratko Djukanović, Paul Brinkman, Johan Kolmert, Cristina Gomez, James Schofield, Joost Brandsma, Andy Shapanis, Paul J. S. Skipp, Anthony Postle, Craig Wheelock, Sven-Erik Dahlen, Peter J. Sterk, Thomas Brown, David J. Jackson, Adel Mansur, Ian Pavord, Mitesh Patel, Christopher Brightling, Salman Siddiqui, Peter Bradding, Ian Sabroe, Dinesh Saralaya, Livingstone Chishimba, Joanna Porter, Douglas Robinson, Stephen Fowler, Peter H. Howarth, Louisa Little, Thomas Oliver, Kayleigh Hill, Louise Stanton, Alexander Allen, Deborah Ellis, Gareth Griffiths, Tim Harrison, Ayobami Akenroye, Jessica Lasky-Su, Liam Heaney, Rekha Chaudhuri, Ramesh Kurukulaaratchy, on behalf of the SoMOSA study team and the U-BIOPRED study team, Ratko Djukanović

**Affiliations:** ^1^ School of Clinical and Experimental Sciences, Faculty of Medicine, University of Southampton and National Institute for Health and Care Research Southampton Biomedical Research Centre, Southampton, United Kingdom;; ^2^ Department of Respiratory Medicine, Amsterdam University Medical Center, University of Amsterdam, the Netherlands;; ^3^ Institute of Environmental Medicine, Karolinska Institutet, and the Department of Respiratory Medicine and Allergy, Karolinska University Hospital, Stockholm, Sweden;; ^4^ Biological Sciences, University of Southampton, Southampton, United Kingdom;; ^5^ Portsmouth Hospitals University National Health Service Trust, Queen Alexandra Hospital, Portsmouth, United Kingdom;; ^6^ Guy’s Severe Asthma Centre, School of Immunology & Microbial Sciences, King’s College London, London, United Kingdom;; ^7^ University of Birmingham and Heartlands Hospital, University Hospitals Birmingham National Health Service Foundation Trust, Birmingham, United Kingdom;; ^8^ Oxford Respiratory National Institute for Health and Care Research Biomedical Research Centre, Nuffield Department of Medicine, University of Oxford, Oxford, United Kingdom;; ^9^ Respiratory Medicine and R&D, University Hospitals Plymouth National Health Service Trust, Plymouth, United Kingdom;; ^10^ Institute for Lung Health and Leicester National Institute for Health and Care Research Biomedical Research Centre, University of Leicester, Leicester, United Kingdom;; ^11^ Clinical Research Facility, Sheffield Teaching Hospitals National Health Service Foundation Trust, Sheffield, United Kingdom;; ^12^ Bradford Institute for Health Research and the National Patient Recruitment Centre, Bradford, United Kingdom;; ^13^ Clinical Sciences, Liverpool University Hospitals National Health Service Foundation Trust, Liverpool, United Kingdom;; ^14^ University College London Respiratory and National Institute for Health and Care Research University College London Hospitals Biomedical Research Centre, London, United Kingdom;; ^15^ Division of Infection, Immunity and Respiratory Medicine, School of Biological Sciences, The University of Manchester, Manchester, United Kingdom;; ^16^ Manchester Academic Health Science Centre and National Institute for Health and Care Research Manchester Biomedical Research Centre, Manchester University Hospitals National Health Service Foundation Trust, Manchester, United Kingdom;; ^17^ Southampton Clinical Trials Unit, University of Southampton, and University Hospital Southampton National Health Service Foundation Trust, Southampton, United Kingdom;; ^18^ Nottingham Respiratory National Institute for Health and Care Research Biomedical Research Centre, University of Nottingham, Nottingham, United Kingdom;; ^19^ Division of Allergy and Clinical Immunology and; ^20^ Channing Division of Network Medicine, Brigham and Women’s Hospital, Harvard Medical School, Boston, Massachusetts;; ^21^ Wellcome-Wolfson Institute for Experimental Medicine, Belfast, Northern Ireland; and; ^22^ Gartnavel General Hospital and School of Infection & Immunity, University of Glasgow, Glasgow, United Kingdom

## Abstract

**Background:**

The anti-IgE monoclonal antibody omalizumab is widely used for severe asthma. This study aimed to identify biomarkers that predict clinical improvement during 1 year of omalizumab treatment.

**Methods:**

One-year open-label Study of Mechanisms of action of Omalizumab in Severe Asthma (SoMOSA) involving 216 patients with severe (Global Initiative for Asthma step 4/5) uncontrolled atopic asthma (at least two severe exacerbations in the previous year) taking high-dose inhaled corticosteroids and long-acting β-agonists with or without maintenance oral corticosteroids. It had two phases: 0–16 weeks, to assess early clinical improvement by Global Evaluation of Therapeutic Effectiveness (GETE); and 16–52 weeks, to assess late responses based on ⩾50% reduction in exacerbations or mOCS dose. All participants provided samples (exhaled breath, blood, sputum, urine) before and after 16 weeks of omalizumab treatment.

**Measurements and Main Results:**

A total of 191 patients completed phase 1; 63% had early improvement. Of 173 who completed phase 2, 69% had reduced exacerbations by ⩾50% and 57% (37 of 65) taking mOCSs had reduced their dose by ⩾50%. The primary outcomes 2,3-dinor-11-β-PGF2α, GETE score, and standard clinical biomarkers (blood and sputum eosinophils, exhaled nitric oxide, serum IgE) did not predict either clinical response. Five volatile organic compounds and five plasma lipid biomarkers strongly predicted the ⩾50% reduction in exacerbations (receiver operating characteristic areas under the curve of 0.780 and 0.922, respectively) and early responses (areas under the curve of 0.835 and 0.949, respectively). In an independent cohort, gas chromatography/mass spectrometry biomarkers differentiated between severe and mild asthma.

**Conclusions:**

This is the first discovery of omics biomarkers that predict improvement in asthma with biologic agent treatment. Prospective validation and development for clinical use is justified.

**At a Glance Commentary**

**Scientific Knowledge on the Subject**

The mechanisms of action of the anti-IgE biologic agent omalizumab in asthma are poorly understood, and commonly measured biomarkers (exhaled nitric oxide, serum IgE, eosinophils) cannot reliably predict the clinical response to treatment. In the age of stratified medicine, the search for reliable ways to predict clinical responses to biologic agents must be extended to the spectrum of omics biomarkers that have transformed our understanding of the mechanisms of asthma.

**What This Study Adds to the Field**

This is the first study to provide proof of concept that omics methods can prospectively identify biomarkers that predict to a high degree whether patients respond to omalizumab based on a ⩾50% reduction in acute exacerbations. This study offers a set of volatile organic compounds as the most promising biomarkers for the prediction of clinical response and a set of plasma biomarkers for which laboratory methods to measure individual biomarkers would be needed. Prospective studies comparing clinical responses in patients selected by these biomarkers with those selected according to criteria used in current practice are needed to validate the candidate biomarkers identified in our study for use in clinical practice.

The anti-IgE monoclonal antibody omalizumab (Xolair) is widely used to reduce asthma exacerbations and the need for oral corticosteroids (OCSs) in severe allergic asthma (1–3), but there is no reliable way to predict its benefit. In current practice, patients with at least two severe exacerbations in the previous year requiring OCSs are given a 16-week therapeutic trial, and the response is assessed using the Global Evaluation of Treatment Effectiveness (GETE) (4), a clinical tool based solely on the physician’s assessment. GETE responders are then advised to continue treatment and undergo review after 1 year of treatment for a reduction in severe acute asthma exacerbations or dose of maintenance OCSs (mOCSs). Although using GETE enriches the responder population (4), a significant proportion of selected patients do not benefit in the long term, and there may be GETE nonresponders who show a response later. Thus, there is an unmet need for predictive biomarkers to optimize the use of omalizumab.

Studies evaluating standard, simple-to-measure clinical biomarkers as predictors of clinical response to omalizumab have had inconsistent results (5); none have assessed biomarker combinations. To improve our understanding of the mechanisms of action of omalizumab and identify predictive biomarkers for clinical practice, we designed a real-world Study of Mechanisms of action of Omalizumab in Severe Asthma (SoMOSA). In this article, the focus is on identifying biomarkers that predict which patients show improvement with treatment. We hypothesized that omics biomarkers (“breathomics,” proteomics, lipidomics) and urine eicosanoids in readily obtained samples (exhaled breath, blood, sputum, urine) can predict early responses (using GETE at 16 wk) and late responses (⩾50% reduction in acute exacerbations or mOCS dose during the first year of treatment), outcomes that are the rationale for prescribing biologic agents. We measured more than 1,400 omics variables developed by the Unbiased Biomarkers Predictive of Respiratory Disease outcomes (U-BIOPRED) program (6, 7), including the prostaglandin D_2_ metabolite 2,3-dinor-11β-PGF2α and LTE_4_ (leukotriene E_4_), whose concentrations we have previously found to be lower in patients with severe asthma treated with omalizumab than in patients receiving standard treatment (8). The predictive value of omics biomarkers was compared with GETE score and standard clinical practice biomarkers (fractional exhaled nitric oxide [Fe_NO_], blood and sputum eosinophil counts, serum IgE). Evidence of the clinical relevance of the identified predictive biomarkers was then sought in datasets from two independent cohorts: U-BIOPRED (6, 7) and the Massachusetts General Brigham (MGB) Biobank (9).

## Methods

### Study Design and Clinical Assessment in the Core SoMOSA Study

This was an open-label, real-world study; all participants received standard-of-care omalizumab and met current inclusion criteria. After 16 weeks of treatment (study phase 1), patients were evaluated by GETE score for early responses. At study end (52 wk), late responses were defined as a ⩾50% decrease in asthma exacerbations or dose of mOCSs between 16 and 52 weeks of treatment (phase 2). Asthma severity and control were assessed using the Asthma Control Questionnaire 7, Asthma Control Test, and Standardized Asthma Quality of Life Questionnaire. In contrast to standard practice, patients considered nonresponders based on the GETE assessment were also invited to continue treatment in phase 2. The study protocol was approved by the Wales Research Ethics Committee 5, Bangor (15-WA-0302), and patients provided written informed consent.

Two independent cohorts, U-BIOPRED and the MGB Biobank, provided data used to seek additional clinical value of any identified predictive biomarkers in SoMOSA.

### Participants

For the core SoMOSA study in patients from 17 tertiary severe asthma clinics, the inclusion criteria were severe asthma (Global Initiative for Asthma step 4/5) that was uncontrolled (Asthma Control Questionnaire score ⩾1.5, atopic, at least two severe exacerbations in the past year) despite high-dose inhaled corticosteroids and long-acting β-agonists with or without mOCSs, serum total IgE concentration 30–1,500 IU/ml, and age 18–70 years (*see* online supplement for complete criteria).

Biomarker datasets from two independent cohorts were identified as suitable for additional analysis of the biomarkers shown in the core SoMOSA cohort as predictive of clinical responses to omalizumab: the U-BIOPRED study (10) and the MGB Biobank (*see* online supplement for details of cohorts and methods).

### Standard and Omics Biomarkers

In the SoMOSA study, patients provided exhaled breath, blood, induced sputum, and morning urine samples before and after 16 weeks of treatment. Four analytical omics methods that are able to quantify large numbers of biomarkers (6, 7) were applied and compared for predictive efficacy with biomarkers often used in clinics (blood and sputum eosinophil counts and Fe_NO_) and with the GETE-based early clinical response tool. Ultra–high-performance liquid chromatography (LC)–tandem mass spectrometry (MS) measured urine concentrations of 14 arachidonic acid–derived eicosanoids (11). Exhaled breath was analyzed by two methods: *1*) gas chromatography (GC)–MS for individual volatile organic compounds (VOCs) and *2*) a combination of electronic nose (eNose) cross-reactive sensors (12) that produced signatures without VOC identities. Intact lipids in sputum and plasma were measured by ultra–high-performance supercritical fluid chromatography–ion mobility–tandem MS (13). Quantitative data-independent LC/HDMS^E^ (liquid chromatography/high-definition mass spectrometry) was used to measure proteins in sputum and morning urine (7).

The omics methods applied in the U-BIOPRED study were broadly the same as those used in SoMOSA, with some technical advances in the latter. Plasma samples from the MGB Biobank underwent global metabolomic profiling (Metabolon) using untargeted LC-MS platforms, which includes amines, amino acids, and polar and nonpolar lipids (14). *See* online supplement for more details of U-BIOPRED and MGB Biobank analytical methods.

### Power Calculation and Statistical Analysis

The change in the urine prostaglandin D_2_ metabolite 2,3-dinor-11β-PGF2α from baseline to 16 weeks after the initiation of omalizumab was the selected as the primary outcome and for power calculation using data from a U-BIOPRED study comparing asthmatic subjects taking and not taking omalizumab (8). Omics biomarkers were prespecified as coprimary outcomes because power calculations are not possible for unbiased omics biomarkers. Assuming 66% of participants would show a response (2:1 ratio of responders to nonresponders), 194 completed participants were required, with sample size adjustment allowed depending on the final ratio of responders to nonresponders. The same calculation was used to compare exacerbation responders and nonresponders. The same participant number was assumed to be required to test the hypothesis that 2,3-dinor-11β-PGF_2α_ in urine would be reduced in participants with a ⩾50% reduction in exacerbations (*see* online supplement for more details).

Initial analysis of treatment effects on patient-reported outcomes, FEV_1_% predicted, Fe_NO_, and blood and sputum eosinophil counts was performed with analysis of covariance or quantile regression models depending on the distribution of the data. For the omics analysis, missing values were addressed as previously described (7, 13), excluding from analysis molecules with detection rates across samples below <40% for proteins and 60% for lipids. Because of differences in methodology between lipidomics and proteomics, missingness was dealt with differently: lipidomics data were imputed using 50% of the lowest limit of detection, whereas, for proteomics, we used median levels to minimize identification of false-positive markers. Data were batch-corrected for location, defining GETE score and exacerbations as outcomes of interest to preserve variation. Features that detected contaminants due to sample collection and/or processing were removed. Data were then split 50/50 into training and test cohorts; the latter was analyzed after a final model was produced on the training cohort. Feature selection was performed on the training data. The equal Gini estimator sought to identify the top five predictive features for each omics platform data set, which were then used to train the final machine learning prediction model using a random forest algorithm, with fivefold cross-validation repeated three times. After training, the prediction model was tested on the test cohort, and the results were plotted as receiver operating characteristic (ROC) curves. Comparisons of identified predictive biomarkers from the core SoMOSA study were made using the U-BIOPRED and MGB Biobank datasets using two-sample Wilcoxon tests applied to participants with severe and mild to moderate asthma in the former and omalizumab responders and nonresponders in the latter. Sparse partial least squares discriminant analysis was applied to the U-BIOPRED data set to assess whether those groups of biomarkers identified by random forest analysis to predict clinical responses could differentiate between those with severe and mild/moderate asthma and between patients taking omalizumab and not taking omalizumab.

## Results

### Analysis of the SoMOSA Study Data

**Figure 1.**
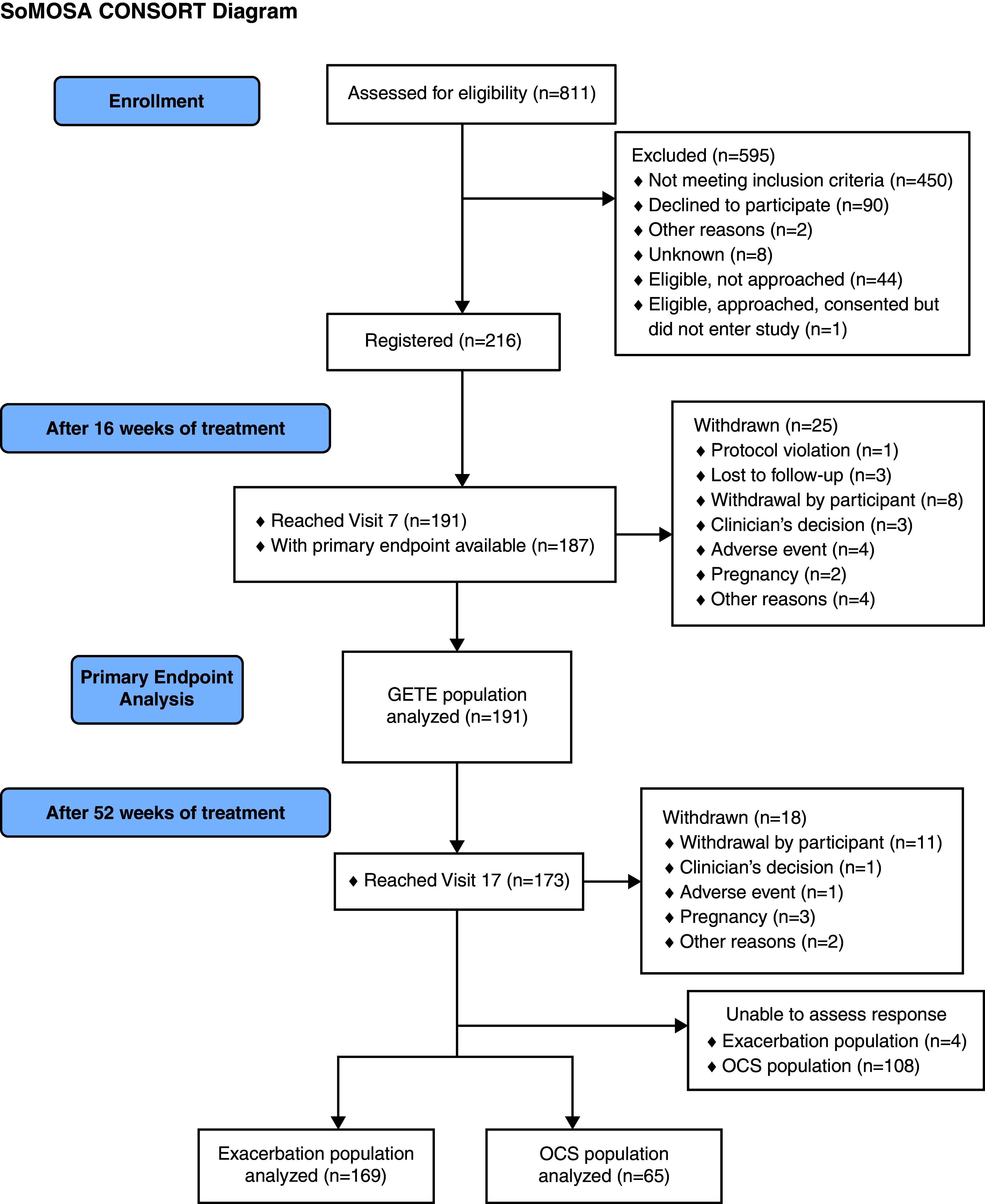
Consolidated Standards of Reporting Trials diagram. OCS = oral corticosteroid.

Of 811 initially assessed patients, 217 were enrolled; 191 successfully completed phase 1, 173 completed phase 2, and 43 withdrew (Figure 1 and Tables E1 and E2 in the online supplement). In keeping with the prespecified allowance to adjust the required number of patients completing the study, recruitment stopped after 191 patients completed phase 1.

#### Clinical responses

Based on GETE score at 16 weeks, 121 of 191 patients (63%) were classified as early responders (Table 1). The majority (*n* = 173; 91%) completed phase 2; of those, 120 (71%) were late responders based on a ⩾50% reduction in acute exacerbation (Table 1) unrelated to age, sex, smoking history, or body mass index (*see* Table E2). Of 65 patients taking mOCSs, 37 (57%) reduced the dose by ⩾50% without losing asthma control (Table 1). Among early responders not taking mOCSs, 71.6% also met late-responder criteria; similarly, 70.7% of late responders not initially taking mOCSs were also early responders. Among patients taking mOCSs at enrolment, 80% of early responders met the criteria for late responders by acute exacerbations or mOCS use. Taking these two late response criteria together, 62% of late responders were also early responders, whereas 63% of early non responders (44 of 70), who would normally be asked to stop treatment, were shown in phase 2 to be late responders based on reduced exacerbations, reduction in mOCS use, or both. Thus, of 36 GETE nonresponders not taking mOCSs, 24 (67%) had a positive response in phase 2. Of the 34 GETE nonresponders using mOCSs before treatment, 20 (59%) had a positive response in phase 2.

**Table 1.** Demographic and Main Clinical Outcomes

	*n*	ACQ7	ACT	AQLQ	AcEx	Taking mOCSs, *n*	IgE, IU	Fe_NO_, ppb	Blood eos, x10^9^/L	Sputum eos, %
Early (16 wk)										
Responders	121 (63.3%)									
Baseline	121	2.9 (2.0–3.6)	12.0 (9.0–17.0)	3.8 (2.9–5.0)	4.0 (3.0–6.0)	41/41 (33.1%)	231.0 (114.0–377.0)	33.5 (17.3–59.0)	0.26 (0.11–0.48)	6.0 (0.9 –23.0)
16 wk	121	1.6 (1.0–2.1)	18.0 (14.0–21.0)	5.5 (4.5–6.2)	NA	41/41 (32.2%)	NA	23.5 (12.8–39.0)	0.18 (0.10–0.38)	1.6 (0.5–8.1)
52 wk	113	1.6 (0.9–2.3)	18.0 (15.0–22.0)	5.7 (4.8–6.3)	1.0 (0.0–2.0)	31/39 (30.6%)	NA	26.0 (16.0–48.5)	0.20 (0.11–0.37)	2.3 (1.0–12.0)
Nonresponders	70 (36.7%)									
Baseline	70	2.9 (2.4–3.7)	10.0 (9.0–13.0)	3.7 (2.9–4.7)	4.0 (2.0–6.0)	34/34 (48.6%)	157.0 (87.0–302.0)	34.3 (16.0–63.0)	0.23 (0.11–0.41)	2.3 (0.9–13.9)
16 wk	70	2.6 (2.0–3.3)	13.0 (10.0–16.0)	4.2 (3.4–5.2)	NA	34/34 (47.1%)	NA	29.8 (19.0–52.0)	0.15 (0.08–0.30)	2.3 (0.6–5.6)
52 wk	60	2.4 (1.1–3.0)	14.5 (10.5–19.0)	4.6 (3.7–5.8)	1.0 (0.0–3.0)	25/29 (40.0%)	NA	23.0 (15.0–41.0)	0.17 (0.07–0.38)	2.5 (0.5–10.0)
AcEx (52 wk)										
Responders	120 (71.0%)									
Baseline	120	2.7 (2.0–3.6)	12.0 (9.0–16.0)	3.8 (3.0–5.0)	4.0 (3.0–6.0)	38/38 (31.7%)	209.8 (112.5–326.4)	33.0 (16.0–56.0)	0.25 (0.11– 0.44)	3.0 (1.0–17.8)
16 wk	120	1.9 (1.1–2.6)	17.5 (14.0–21.0)	5.2 (4.1–6.1)	NA	38/38 (30.0%)	NA	24.0 (14.0–37.0)	0.19 (0.10–0.35)	1.8 (0.5–5.8)
52 wk	120	1.6 (0.9–2.4)	18.5 (13.5–22.0)	5.6 (4.6–6.3)	1.0 (0.0–2.0)	29/38 (30.0%)	NA	22.5 (15.5–42.0)	0.20 (0.10–0.39)	2.3 (0.8–12.3)
Nonresponders	49 (29.0%)									
Baseline	49	3.0 (2.3–3.7)	10.0 (8.0–13.0)	3.8 (2.9–4.8)	4.0 (2.0–5.0)	26/26 (53.1%)	191.7 (98.0–360.0)	31.3 (18.0–53.0)	0.22 (0.11–0.50)	7.8 (1.3–30.5)
16 wk	49	2.4 (1.6–3.0)	13.0 (11.0–18.0)	4.8 (3.7–5.7)	NA	26/26 (51.0%)	NA	28.0 (17.5–51.0)	0.12 (0.09–0.30)	2.3 (0.8–8.5)
52 wk	49	2.4 (1.4–3.0)	14.0 (12.0–18.0)	4.6 (3.9–5.6)	3.0 (2.0–4.0)	24/26 (51.0%)	NA	31.0 (16.5–56.5)	0.18 (0.10–0.38)	2.9 (1.0–6.1)
mOCS (52 wk)										
Responders	37 (56.9%)									
Baseline	37	2.4 (2.0–3.6)	13.0 (10.0–19.0)	4.0 (3.2–5.5)	3.0 (2.0–4.0)	37/37 (100%)	177.0 (87.0–492.0)	36.0 (19.0–55.5)	0.24 (0.09– 0.37)	3.9 (1.5–12.5)
16 wk	37	2.0 (1.3–2.6)	18.0 (13.0–21.0)	5.1 (4.4–6.1)	NA	37/37 (100%)	NA	30.0 (17.5–44.0)	0.16 (0.07–0.30)	3.5 (0.5–13.5)
52 wk	37	2.0 (0.9–2.9)	17.0 (13.0–19.0)	5.2 (4.6–6.1)	1.0 (0.0–2.0)	23/37 (62.2%%)	NA	31.0 (19.5–63.0)	0.25 (0.16–0.42)	7.5 (1.0–21.0)
Nonresponder	28 (43.1%)									
Baseline	28	3.0 (2.4–3.9)	11.0 (9.0–17.0)	4.0 (3.2–4.6)	4.0 (2.0–6.0)	28/28 (100%)	152.0 (101.0–254.0)	30.0 (15.8–72.0)	0.13 (0.06– 0.26)	11.0 (0.8–13.0)
16 wk	28	2.4 (1.1–3.5)	15.5 (10.5–21.5)	4.9 (3.5–6.0)	NA	28/28 (100%)	NA	27.5 (20.8–61.8)	0.10 (0.04–0.26)	2.6 (1.5–8.5)
52 wk	28	2.3 (1.4–3.9)	16.0 (10.5–22.0)	4.6 (3.5–6.1)	2.0 (1.0–3.5)	28/28 (100%)	NA	27.0 (15.5–63.5)	0.12 (0.06–0.32)	4.9 (1.3–24.8)
*P* values*										
16 wk		<0.001	<0.001	<0.001	NA	NA	NA	0.014	0.180	0.227
52-wk AcEx		0.005	0.002	0.001	NA	NA	NA	0.077	0.099	0.344
52-wk mOCS		0.479	0.853	0.442	NA	NA	NA	0.504	0.166	0.251

*Definition of abbreviations*: AcEx = acute exacerbation; ACQ = asthma control questionnaire; ACT = asthma control test; AQLQ-S = asthma quality of life questionnaire; Fe_NO_ = fractional exhaled nitric oxide; eo = eosinophil; mOCS = maintenance oral corticosteroid.

Data in parenthetical ranges are IQRs. Model: comparison of changes in variable values from baseline to 16 or 52 weeks, with variable after 16 of the 52 weeks of treatment = intercept + response group + variable at baseline. The numbers of participants who enrolled and remained in the study at 16 and 52 weeks are shown in the second column. The mOCS use data are shown as the numbers of participants taking a mOCS at the time of assessment as a proportion of the total numbers of patients assessed (i.e., still in the study) at that time point.

* *P* values determined by analysis of covariance/quantile regression depending on the distribution of the data.

The numbers of responders and nonresponders based on exacerbation reduction (120 and 49, respectively) or GETE score (121 and 70, respectively) were deemed sufficient to split the cohort into training and test sets. In contrast, the numbers of responders by mOCS reduction (37 and 28, respectively) were too small for analysis.

#### Biomarker measurements

A total of 1,408 variables passed quality control. Because individual biomarker molecules can result in multiple MS variables that require deconvolution to produce single variables, the 1,408 variables were reduced to 14 eicosanoids, 70 breath VOCs, 112 sputum proteins, and 147 urine proteins. A further 158 eNose variables provided signatures without molecular identities. Of the 589 lipid variables in plasma and 305 in sputum, identities were determined only if concentrations were different between responders and nonresponders (86 in plasma and 25 in sputum).

#### Baseline differences in biomarkers between responders and nonresponders

**Figure 2.**
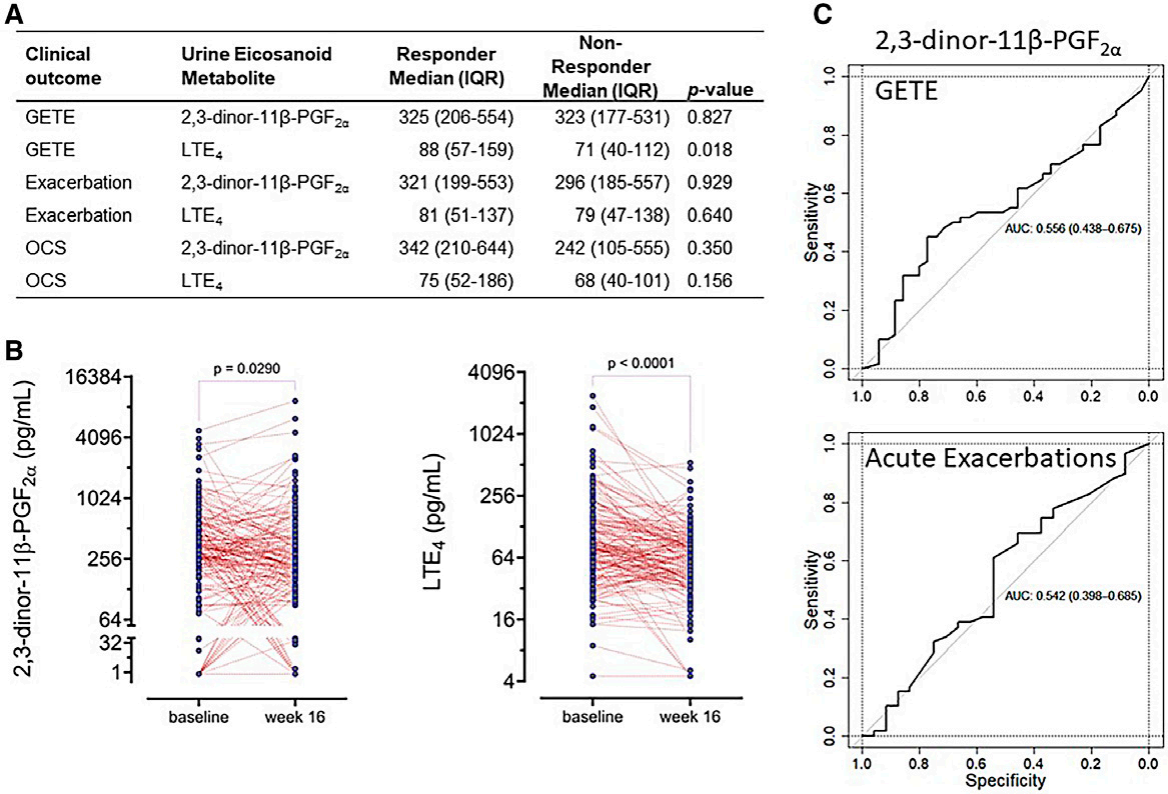
Urine eicosanoids, 2,3-dinor-11-β-PGF_2α_, and LTE_4_ (leukotriene E_4_). (*A*) Baseline concentrations of 2,3-dinor-11β-PGF2α (primary outcome) and LTE_4_ (pg/ml) in patients defined as responders or nonresponders based on Global Evaluation of Therapeutic Effectiveness score. (*B*) Changes in concentrations of 2,3-dinor-11-β-PGF_2α_ and LTE_4_ in the entire cohort (responders and nonresponders) from baseline to 16 weeks analyzed by Mann-Whitney *U* test. (*C*) Receiver operating characteristic area under the curve for 2,3-dinor-11β-PGF2α in respect to the prediction of early (Global Evaluation of Therapeutic Effectiveness–based) and late (acute exacerbation–based) response to omalizumab. AUC = area under the curve; GETE = Global Evaluation of Therapeutic Effectiveness; OCS = oral corticosteroid.

**Figure 3.**
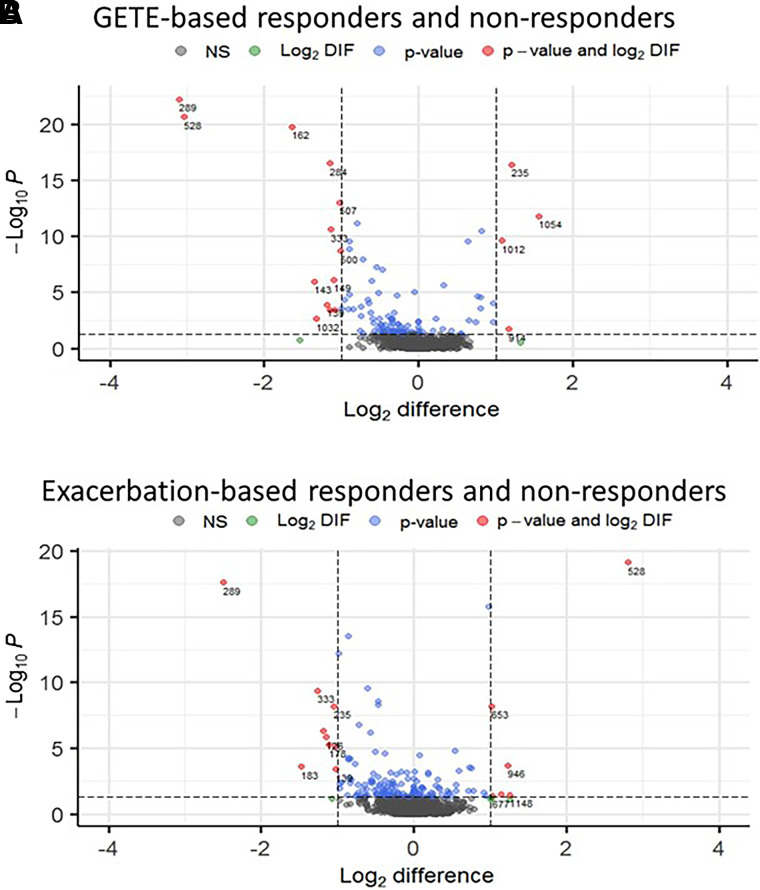
Volcano plots of baseline concentrations of all biomarker variables in responders and nonresponders. Responses shown include early response judged by GETE response (*A*) and late response defined by ⩾50% reduction in exacerbations (*B*). The red and blue biomarkers (all *P* < 0.05) are labeled by numbers (*see* Table E4 for identities). Green and red dots represent greater than onefold different biomarkers. The data are shown as the means of concentrations in the responders from which the means of the concentrations in the nonresponders have been subtracted (i.e., responder minus nonresponder). They are shown as log2-transformed data. The *P* values were obtained by Mann-Whitney *U* test. GETE = Global Evaluation of Therapeutic Effectiveness.

Baseline concentrations of 2,3-dinor-11β-PGF_2α_ (primary outcome) did not differentiate early or late responders and nonresponders (Figure 2 and Figure E1). Even though baseline LTE_4_ was significantly (*P* = 0.018) higher in early responders, LTE_4_ and other eicosanoid levels did not differentiate late responders and nonresponders (Figure 2). The same was true for Fe_NO_, blood and sputum eosinophil counts, and IgE (Table 1). In contrast, a total of 368 omics variables were different between responders and nonresponders across the four omics platforms (Figure 3): 103, 143, and 122 when comparing responses by GETE, exacerbation reduction, and mOCS use reduction, respectively, 67 being different for more than one outcome.

#### Prediction of clinical responses to omalizumab by random forest analysis

The 2,3-dinor-11β-PGF_2α_ did not predict the early GETE-based response (ROC AUC, 0.556) or⩾50% exacerbation reduction during phase 2 (ROC AUC, 0.542) (Figure 2), nor did the other urine eicosanoids (data not shown). Similarly, GETE, Fe_NO_, blood or sputum eosinophil counts, and serum IgE (Figure E2) did not predict exacerbation reductions.

**Figure 4.**
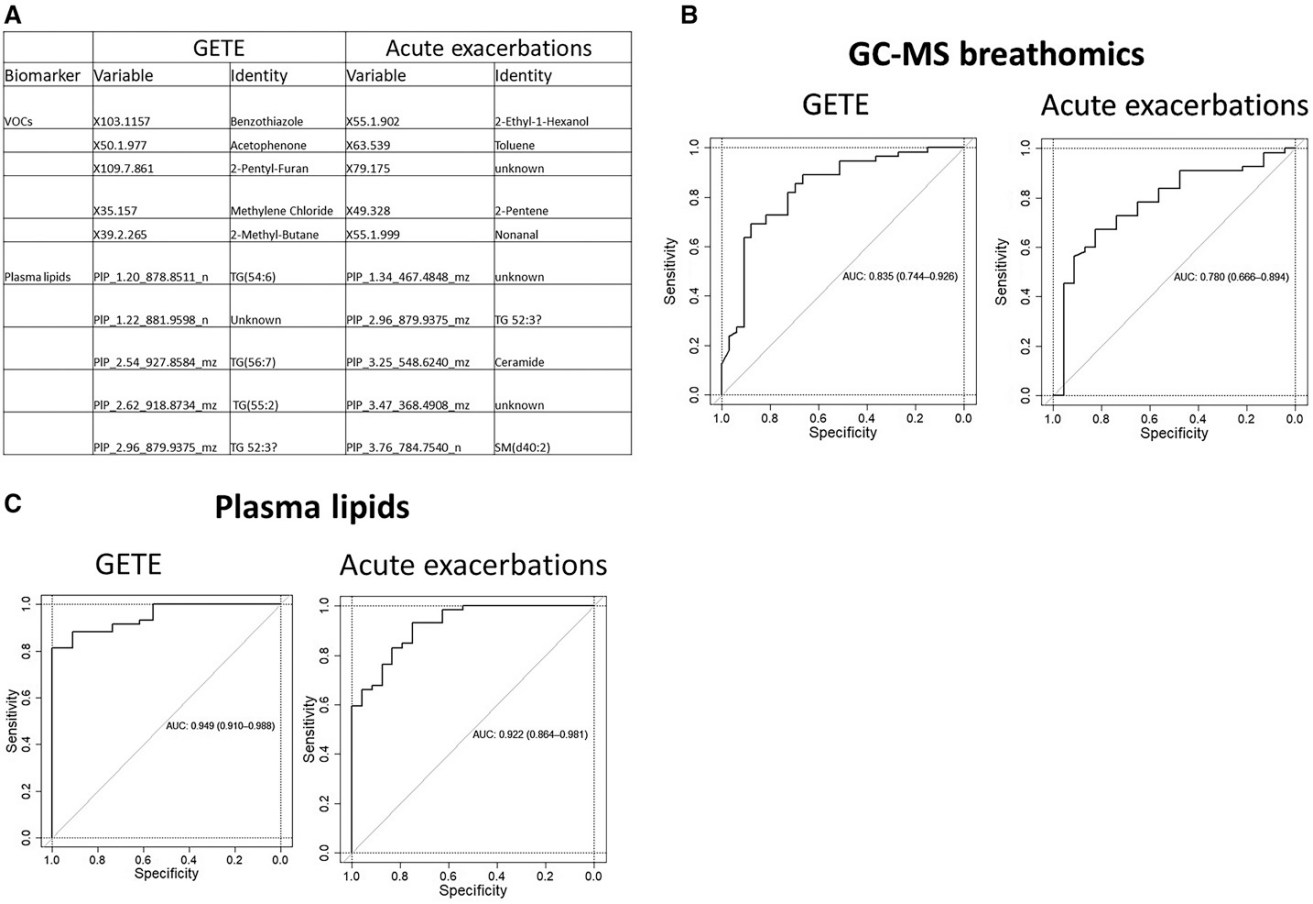
Breath volatile organic compounds (VOCs) and plasma lipids that predict early or late clinical responses. (*A*) The biomarker identities of the VOCs were derived from the variables detected by gas chromatography–mass spectrometry, whereas the identities of the plasma lipids were derived from the variables detected by ultra–high-performance supercritical fluid chromatography–ion mobility–tandem mass spectrometry. Receiver operating characteristic area under the curve figures show predictions by VOCs (*B*) and lipids (*C*) of early clinical responses judged by GETE score and late responses by reduction in asthma exacerbations. *See* Table E3 for the receiver operating characteristic area under the curve values for the other omics platforms (sputum lipids, sputum proteins, urine proteins, and eicosanoids). AUC = area under the curve; GETE = Global Evaluation of Therapeutic Effectiveness

Analysis of all the omics platforms showed that breathomics and plasma lipidomics predicted early and late responses (Figure 4), whereas the other omics platforms had weak predictive value (Table E3). One set of five exhaled breath VOCs (benzothiazole, acetophenone, 2-pentyl-furan, methylene chloride, 2-methyl-butane) predicted early improvement (ROC AUC, 0.835); another set of VOCs (2-ethyl-1-hexanol, toluene, 2-pentene, nonanal, and a VOC of unknown identity detected as X79.175 by GC-MS) predicted a ⩾50% exacerbation reduction (ROC AUC, 0.780). Two sets of five plasma lipids were highly predictive of early and late clinical responses (ROC AUCs, 0.949 and 0.922, respectively). The plasma lipids that predicted early responses consisted of four triglycerides (TG[54:6], TG[56:7], TG[55:2], and TG[52:3]) and a currently unidentified lipid. A further set predicted exacerbation reductions; of these, only one could be identified in lipid databases or the wider literature, namely the sphingomyelin peak for SM(d40:2), likely comprising a combination of SM(d18:2/22:0), SM(d16:1/24:1), and SM(d18:1/22:1) molecular species (15). Two further peaks were putatively identified as TG52:3 and ceramide.

#### Effect of treatment on eicosanoids and standard biomarkers

Urinary 2,3-dinor-11-β-PGF_2α_ decreased significantly (*P* = 0.029) after 16 weeks of treatment, with no difference between responders and nonresponders (Figure 2). LTE_4_ was also reduced (*P* < 0.001), but to a similar extent in responders and nonresponders (Figure 2). The other urine eicosanoids did not change (data not shown).

In the entire cohort, omalizumab reduced blood and sputum eosinophil numbers during phase 1 (*P* < 0.001 and *P* < 0.023, respectively) and Fe_NO_ and blood eosinophils during phase 2 (*P* = 0.022, *P* < 0.001, respectively), but these changes were not related to treatment responses except for Fe_NO_, which was reduced more in early responders (*P* = 0.014); however, neither Fe_NO_ nor any of the other standard biomarkers discriminated late responders and nonresponders by ROC analysis in isolation or when combined (*see* Figure E2). We also stratified patients according to Fe_NO_ and blood eosinophil count cutoff values used by Hanania and colleagues (2) as biomarker-high or -low when assessing their clinical response to omalizumab. We found that such stratification did not predict which stratum of patients would respond to omalizumab (Figure E3). Similarly, time to the first protocol-defined asthma exacerbation, as demonstrated by Kaplan-Meier curves, was no different (Figure E4) between these strata of patients.

#### Analysis of the identified predictive biomarkers in the U-BIOPRED and MGB Biobank

A search of the U-BIOPRED data undertaken for matching VOCs and plasma lipids showed that several of the candidate biomarkers we found to be predictive of responses to omalizumab were able to differentiate between individuals with severe atopic asthma and mild/moderate asthma (*see* online supplement for full details) in the U-BIOPRED cohort. In the MGB Biobank, the concentrations of plasma sphingomyelin (sphingomyelin d18:1/22:1, d18:2/22:0, d16:1/24:1) were significantly (*P* = 0.03) lower in responders to omalizumab than in nonresponders.

## Discussion

To our knowledge, this is the first study to use a multi-omics approach to identify predictive biomarkers for severe asthma, providing proof of concept that breathomics and plasma lipidomics biomarkers can predict who benefits from omalizumab during the first 16 weeks of treatment and who shows a ⩾50% reduction in exacerbations during the first year of treatment. In an independent cohort, the biomarkers identified in SoMOSA were shown to differentiate between mild/moderate and severe asthma, including those with more frequent exacerbations who would be candidates for treatment with omalizumab. Development of these biomarkers has significant potential to give patients, their medical teams, and payers more certainty of achieving reduced exacerbations with omalizumab, a key objective of asthma treatment.

Consistent with previously reported efficacy, 63% of patients showed improvement within 16 weeks of starting treatment, which suggests that the enrolled cohort is representative of the typical patient considered for omalizumab. In our study, GETE, the clinical tool widely used to assess clinical response to omalizumab, did not predict late improvement (*see* Figure E2); indeed, many patients classified by GETE as non–early responders had a late response (i.e., reduced exacerbations or mOCS use). Although 2,3-dinor-11B-PGF2a, the coprimary outcome used for power calculation, was reduced significantly with treatment, the changes were similar in responders and nonresponders, and baseline concentrations did not predict early or late improvement (Figure 2). Similarly, none of the standard biomarkers currently used in asthma management (Fe_NO_, sputum and blood eosinophil counts, and serum IgE) had predictive value (*see* Figure E2).

Breathomics is a growing field in medicine (16). There are several types of eNoses that provide signatures, but not identities of VOCs, and MS methods like GC-MS effectively predict clinical and therapeutic outcomes. Whereas the combination of eNose cross-reactive sensors could not predict clinical improvement, five VOCs (2-ethyl-1-hexanol, toluene, 2-pentene, and one unknown VOC) derived by GC-MS confidently predicted the reductions in exacerbations, whereas a separate set of five GC-MS–derived VOCs (benzothiazole, acetophenone, 2-pentyl-furan, methylene chloride, and 2-methyl-butane) predicted good early responses. Together, these VOCs differentiated between individuals with mild/moderate asthma and atopic severe asthma (ROC AUC, 0.931) and between mild/moderate asthma and severe asthma prone to exacerbations (at least two exacerbations per year), a cutoff for initiating treatment with a biologic agent. Many of these VOCs have been reported in respiratory studies. Nonanal is associated with neutrophilic asthma and smoking; it has been able to predict exacerbations and discriminate between allergic and nonallergic asthma in children (17–19). Toluene, a common organic solvent, is increased in smokers (20), is related to environmental exposure (21), and has also been associated with asthma (22). We have previously found nonanal within a group of exhaled breath biomarkers in patients with cystic fibrosis with sputum positive for *Pseudomonas aeruginosa* (23). The predictive set in our study also included 2-ethyl-1-hexanol, for which there is prior evidence of a role in asthma and in lung cancer (reviewed by Sola-Martinez and coworkers [24]). It is a known indoor pollutant and the main metabolite of di(2-ehylhexyl)phthalate, a solvent and frequent plasticizer of polyvinylchloride. Concentrations of 2-ethyl-1-hexanol sampled in ambient air are negligible compared with those in exhaled breath (24), suggesting that, if it is in part inhaled, it is concentrated in the lungs. 2-ethyl-1-hexanol is produced in greater quantities by cancer cells (25). Within the lungs, it acts as an endocrine-disrupting chemical and is associated with oxidative stress and modulation of immune responses (26). The hydrocarbon 2-pentene, also a solvent and known byproduct of thermal cracking of petroleum, is found in ambient air. It is also a volatile compound derived from lipid peroxidation, with increased concentrations found by GC-MS in the headspace of bacterial cultures (27). Among the GC-MS variables that predicted early improvement, three have been reported in respiratory conditions: acetophenone in patients with cystic fibrosis with *Pseudomonas aeruginosa* (23) and 2-pentylfurane in patients with *Aspergillus fumigatus* (28). Analysis of VOCs in exhaled breath that diagnose ventilator-associated pneumonia has proposed a set of 12 predictive VOCs, among them 2-methyl-butane (29). We could not find any similar reports for benzothiazole.

Lipidomic analysis of plasma also identified two sets of predictive biomarkers. Early improvement was predicted by four triglycerides and one unknown lipid species. In comparison to our understanding of the roles of leukotrienes, knowledge of other lipids in asthma is limited, although obesity is strongly associated with asthma. Serum triglyceride levels are higher in obese people with asthma, even when adjusted for body mass index, blood eosinophils, and statin treatment (30). A recent lipidomics study that identified more than 1,300 plasma lipid species showed that triglyceride levels, albeit different from the ones in our analysis, differentiated asthma from health and were related to asthma severity (31), with ceramides being related to asthma severity, in keeping with the findings in our study. Ceramide exacerbates inflammation, mucus production, and endoplasmic reticulum stress, and increased levels are associated with airway hyperresponsiveness, a key feature of asthma (32). However, these lipids were not good at differentiating between severe and mild/moderate asthma and asthma with frequent exacerbations in the U-BIOPRED study; even though concentrations of plasma triglyceride 52:3 and one unidentified lipid were significantly higher in those with severe atopic asthma and in those with at least two exacerbations per year, the ROC AUC indicated weak differentiation (*see* online supplement). Of note, however, comparison of responders and nonresponders to omalizumab (defined by ⩾50% reduction in exacerbations) in the MGB cohort showed significantly lower concentrations of sphingomyelin (d18:1/22:1, d18:2/22:0, d16:1/24:1) in responders.

This study has limitations. It could be argued that we should have used a classical randomized controlled trial design, despite ample precedent of similar study design in oncology. Our discussions with the patient advisory group strongly favored a real-world study design, arguing that a placebo arm would be unethical because it would deny patients a drug known to improve a severe condition and that recruitment into a placebo-controlled trial would be difficult because omalizumab is readily available and patients expect to be treated. The fact that study recruitment took 26 months and required engagement of 17 severe asthma centers with exclusive rights to prescribe biologic agents justified this decision. The other limitation of the study is that there were too few patients in whom mOCS treatment was reduced by ⩾50%, a measure that is very relevant to patients because of OCS side effects.

The identified biomarkers should be viewed as candidate biomarkers that require confirmation in a prospective study in which treatment efficacy in patients selected based on these biomarkers would be compared with efficacy in patients selected based on standard clinical criteria. Further studies are also needed to elucidate how these biomarkers are involved in asthma pathogenesis. Prospective validation of the candidate biomarkers should focus on breathomics, an easy-to-apply platform, possibly in combination with plasma lipid measurements. In view of the cost of developing routine analytical methods, the development of single-platform assays is likely to be easier, more acceptable to patients, and less expensive. Although lipids had greater predictive power (AUC >0.9) than the VOC biomarkers (AUCs, 0.835 for early and 0.780 for late responses), breathomics is, in our view, a superior omics platform because of easier sample collection, more certainty about the VOC identities and, most importantly, easier development of point-of-care instruments for clinical use. Further elucidation of the detected lipids would likely be more complex and costly, and, with uncertain outcomes, riskier.
